# ‘Poly phenolic phytoceutical loaded nano-bilosomes for enhanced caco-2 cell permeability and SARS-CoV 2 antiviral activity’: in-vitro and insilico studies

**DOI:** 10.1080/10717544.2022.2162157

**Published:** 2023-01-01

**Authors:** Mohamed Y. Zakaria, Shady M. Abd El-Halim, Botros Y. Beshay, Islam Zaki, Mohammed A.S Abourehab

**Affiliations:** a Department of Pharmaceutics and Industrial Pharmacy, Faculty of Pharmacy, Port Said University, Port Said, Egypt; b Department of Pharmaceutics and Industrial Pharmacy, Faculty of Pharmacy, King Salman International University (KSIU), South Sinai, Ras Sudr, Egypt; c Department of Pharmaceutics and Industrial Pharmacy, Faculty of Pharmacy, October 6 University, 6th of October City, Giza, Egypt; d Pharmaceutical Sciences (Pharmaceutical Chemistry) Department, College of Pharmacy, Arab Academy for Science, Technology and Maritime Transport, Alexandria, Egypt; e Pharmaceutical Organic Chemistry Department, Faculty of Pharmacy, Port Said University, Port Said, Egypt; f Department of Pharmaceutics Faculty of Pharmacy, Umm Al-Qura University, Makkah, Saudi Arabi

**Keywords:** Resveratrol, PEGylated bilosome, Caco-2 cell permeation, anti SARS-CoV-2, docking

## Abstract

Severe acute respiratory syndrome coronavirus-2 (SARS-CoV-2) predisposed to the emergence of worldwide catastrophe that impels the evolution of safe and effective therapeutic system. Polyphenols as resveratrol (RSV) exhibit a well evidenced antiviral activity. Unfortunately, like most phenolic nutraceuticals, RSV suffers from restrained solubility and massive degradation in GIT and liver which in turn prohibit its clinical use. Herein, PEGylated bilosomes (PBs) contain PEGylated edge activator along with the traditional components as (Span 60, cholesterol and bile salts) were proposed to boost both permeability and bioavailability of RSV. The investigation of the prominent effect of the diverse variables on the characteristics of the vesicles and picking of the optimum formula were conducted via construction of 2^3^ factorial experiment. The appraisal of the formulae was conducted on the basis of entrapment efficiency percent (EE%), particle size (PS) and zeta potential (ZP). In addition, the spherical shaped optimal formula (F5) exhibited EE% of 86.1 ± 2.9%, PS of 228.9 ± 8.5 nm, and ZP of −39.8 ± 1.3 mV. The sorted optimum formula (F5) exhibited superior dissolution behaviors, and boosted Caco-2 cells cellular uptake by a round 4.7 folds relative to RSV dispersion. In addition, F5 demonstrated a complete in vitro suppression of SARS-CoV-2 at a concentration 0.48 μg/ml with 6.6 times enhancement in antiviral activity relative to RSV dispersion. The accomplished molecular modeling heavily provided proof for the possible interactions of resveratrol with the key residues of the SARS-CoV2 Mpro enzyme. Finally, F5 could be proposed as a promising oral panel of RSV for curation from SARS-CoV-2 infection.

## 1. Introduction

Betacorona virus is the causative microorganism for the latest outbreak of coronavirus disease 2019 (COVID-19), whereas, the International Committee on Taxonomy of Viruses also identified it as severe acute respiratory syndrome coronavirus 2 (SARS-CoV-2). This virus can be also categorized under the enveloped positive-sense single-stranded RNA viruses, unfortunately, it is a highly contagious virus with high transmission rate especially from human to human (Chan et al., 2020). According to the World Health Organization (WHO) reports, the virus leaded to millions of assured COVID-19 severe infections accompanied by high mortality rate by the end of 2020, announcing the emergence of catastrophic and life threatening disease that endanger the world health. Therefore, all the health care research teams all over the world investing massive efforts in order to develop safe and effective drug or vaccine for this drastic disease. Worldwide, many repurposed and off-label medications have been involved in treatment of many of the infected cases as lopinavir-ritonavir, remdesivir, chloroquine, hydroxychloroquine, favipiravir, nitazoxanide, convalescent plasma and azithromycin (Mahmoud, 2020); however, the development of effective therapeutic molecules that possess the ability to resolve COVID-19 becomes a critical and urgent challenge for all researchers (Xu et al., 2020).

The development of new drug candidates and identification of the possible targets involved in treatment essentially depend on various computational approaches as molecular docking studies. It is well known that main protease (Mpro) is in charge of the replication and transcription of the SARS-CoV-2 via the transformation of the polypeptides into functional proteins(McIntosh and Perlman, 2015; Voss et al., 2020). Thus, the elaboration of drug candidates that possess the ability of targeting Mpro of this virus was claimed to be prosperous antivirals. Lately, the use of phytopharmaceuticals as polyphenolic herbal drugs gasps great attention owing to their vast therapeutic activities especially the antiviral one. Unfortunately, their pharmaceutical utilization is hindered attributed to their low solubility and diminished oral bioavailability. So that, the design of proper drug delivery system for these phytoceuticals is the way to gain their therapeutic effectiveness and conquer the previously mentioned limitations.

Resveratrol (RSV) is a well-known phytoceutical compound related to the polyphenols stilbenoids group. Stunningly, it offers interesting and multiple therapeutic and biological applications (Salehi et al., 2018). Among the aforementioned applications, RSV is able to exert a well evidenced anti-tumor activity (Ko et al., 2017), in addition, RSV exhibits anti-inflammatory activity, potent antioxidant properties and anti-aging activity (Camins et al., 2009; Zhou et al., 2018). Even more, it was previously recorded that RSV exhibited a potent anti-viral activity against both DNA and RNA viruses (Lin et al., 2017; Pasquereau et al., 2021; Zakaria et al., 2022b). However, as polyphenolic compounds, RSV suffers from poor aqueous solubility and extensive degradation in GIT and liver that render its bioavailability and therapeutic activities diminished (Poonia et al., 2020).

The commonly used vesicular systems as liposomes and niosomes are employed as nano-carriers for drug encapsulation. Unluckily, their diminished stability encapsulation efficiency along with the problems associated with their scaling up obliges the elaboration of novel vesicular carriers. Bile salts (BS) involvement in the vesicular structure allow the attainment of vesicles exhibiting greater stability relative to liposomes and niosomes; releasing bile salt modified vesicular system identified as Bilosomes (Bs) (Aburahma, 2014). Accordingly, owing to their greater stability and ability to counter act the negative effects and drastic circumstances that face the drugs in GIT as bile salts, enzymes and pH, bilosomes can be successfully used as potential carrier for oral administration of sparsely water soluble drugs and vaccines (Aburahma, 2014; Zakaria et al., 2021). Herein, Brij® was employed in the formulation of the vesicles, which act as extra edge activator (EA) to develop the PEGylated bilosomes (PBs) (Deng et al., 2022). The idea behind the utilization of PEGylated EA namely Brij® which composed of different acyl chain moieties along with various PEG chain lengths (Tagami et al., 2011). It was well reported that the existence of the hydrophilic PEG moieties in EAs could augment the vesicular stability, prolongation of the systemic circulation duration and aid in evading the massive degradation due to first pass metabolism (Jain et al., 2008).

Thus, in the current study, PBs were employed as a delivery system for improving the intestinal permeation, bioavailability and antiviral activity of RSV. For achieving this goal, 2^3^ full experimental design was implemented for studying the significance of the alteration in the fabrication aspects and selection of the optimum formula. Moreover, the optimum formula was involved in further assessments compared to RSV dispersion as in vitro release study and Caco-2 cells cellular uptake to appraise the enhancement in the intestinal permeation. Furthermore, in vitro evaluation of antiviral activity of optimum formula compared to that of RSV dispersion against SARS-CoV-2 was conducted. Finally, molecular docking studies will be conducted to explain the obtained antiviral activity at molecular level.

## 2. Material and methods

### 2.1. Materials

Sigma–Aldrich Chemical Co. (St. Louis, Missouri, USA) was the source of Resveratrol (RSV) and Span 60, while ITX Biomedicals (Santa Ana California, USA) was the source of Cholesterol. Sodium glycholate (SGC) and Sodium deoxycholate (SDC) were purchased from BASF Co. (Florham Park, New Jersey, USA). Absolute ethyl alcohol sodium hydroxide and potassium dihydrogen orthophosphate were acquired from El-Nasr Chemical Co., Cairo, Egypt. SERVA Electrophoresis GmbH., Heidelberg, Germany was the source of (12,000–14,000 molecular weight cut off) VISKING® dialysis membrane. All chemicals and solvents were of analytical grade and were used as received.

### 2.2. Methodology

#### 2.2.1. Experimental design for the formulation of RSV-PEGylated bilosomes (RSV-PBs)

The investigation of vast fabrication aspects of RSV-loaded PEGylated bilosome was conducted via 2^3^ factorial analyses utilizing Design Expert® software version 13 (Stat Ease, Inc., Minneapolis, MN, USA). The design resulted in eight runs considering the investigated factors: bile salt type at 2 levels (A- SGC or SDC), edge activator type and amount (EA type and amount) (B- Brij 20 or Brij 72) and (C-15 mg or 30 mg) respectively, meanwhile EE% (Y1), PS (Y2) and ZP (Y3) were picked as the dependent variables.

#### 2.2.2. Fabrication of RSV loaded PBs

Ethyl alcohol injection technique was employed in fabrication of RSV loaded PBs (Mosallam et al., 2021). In a water bath adjusted at 60 °C, ethyl alcohol was involved in dissolving each of (25 mg) cholesterol, (20 mg) of RSV and (150 mg) Span 60. In 10 ml phosphate-buffered saline (PBS, pH 7.4) containing previously dissolved edge activator and bile salt, the clear organic phase was then injected slowly and the system was kept under continuous stirring. After the appearance of turbidity, the dispersion was continually stirred till complete dispelling of ethyl alcohol. Finally, the resulted dispersions were subjected to bath sonication in a water-bath sonicator (Type USR3, Julabo Labortechnik, Seelbach, West Germany) at 25 °C for PS reduction. The fabricated PBs were preserved in dark tubes at 4 °C for further investigations.

#### 2.2.3. In vitro characterization and optimization of RSV loaded PBs

##### 2.2.3.1. Entrapment efficiency (EE%)

The extent of RSV entrapped within the vesicles was assessed using indirect technique for computing the free (unentrapped) RSV (Abdelbary and AbouGhaly, 2015). In cooling centrifuge (Sigma 3–30 KS, Germany), one milliliter of each PB was centrifuged for 1 hr at 4 °C and speed 20000 rpm. Then the supernatant was diluted and assessed for determination of RSV concentration utilizing UV/Vis spectrophotometer at λmax = 290 nm (Shimadzu, model UV-1601 PC, Kyoto, Japan) (Negi et al., 2017). RSV EE% was calculated by the use the equation as following:

$$\mathrm{EE}\%=\frac{\text{Total amount of RSV}-\text{Unentrapped RSV }}{\text{Total amount of RSV}}\times \;100$$

##### 2.2.3.2. Assessment of RSV loaded PBs PS, PDI, and ZP

After dilution of 0.1 ml of each formula by 10 ml deionized distilled water, the average PS, PDI, and ZP were explored using Zetasizer 2000 (Malvern Instrument Ltd., UK). Each sample was measured three times and the results were represented as the mean value.

#### 2.2.4. Optimization of the prepared RSV-loaded PBs

According the highest EE% and ZP values together with minimum PS the election of the optimum formula was employed using the software Design Expert® version 13. Additionally, the ANOVA assessments were involved in figuring out and investigation of the prime consequences of the studied variables on the pre-determined responses, where the significance of each variable was explored and based on the highest desirability values the optimal formula was elected for further assessments (Aldawsari et al., 2021).

#### 2.2.5. In vitro analysis of the optimized RSV loaded PBs

##### 2.2.5.1. Lyophilization of optimized PBs formula

The optimized RSV-loaded PBs solidification was employed utilizing lyophilizer (Alpha 2–4, CHRIST, Osterodeam Harz, Germany) and for the prohibition of the vesicular lysis and aggregation, mannitol (5% w/v) was incorporated. At 80 °C the dispersion of optimum formula was preserved for overnight succeeded by under vacuum drying for 24 h (Dubey and Vyas, 2021).

##### 2.2.5.2. X-ray diffraction

The measurement of the extent of crystallinity of pure RSV, plain lyophilized RSV-PBs and RSV-loaded PBs was employed via XRD technique using x-ray diffractometer (Burker, Germany) with a Cu Ka radiation detector. The scan was performed in 2 θ range of 3.0°–40° at a scan rate of 1 min.

##### 2.2.5.3. Transmission electron microscopy (TEM)

The surface properties along with the morphology of the selected RSV-PBs optimum formula were explored by using transmission electron microscopy (Joel JEM 2100, Tokyo, Japan). A droplet of formula dispersion was negatively stained using 1% phosphotungstic acid succeeded by copper coating carbon grid, after that this was self-dried to attain a thin film. TEM was then used to scan the copper sheet.

##### 2.2.5.4. In vitro drug release studies

For a comprehensive view on the release pattern of RSV from the fabricated RSV loaded PBs compared to RSV suspension (20 mg of RSV in 10 ml PBS) dialysis technique was employed (Poonia et al., 2020). The cellulose membrane bags containing either 1 ml of the optimum formula after being diluted (1 ml crude formula in 1 ml phosphate buffer) or 1 ml of RSV suspension after being diluted in the same manner both equivalent to 1 mg of RSV were placed in 200 ml (pH 7.4) phosphate-buffered saline. The system was kept under stirring at 100 rpm and the temperature was preserved at 37 °C. One ml sample at predetermined time intervals was taken and equally replenished with fresh buffer to provide a proper sink condition for diffusion. Finally, the samples were analyzed for determination of the drug concentration utilizing UV/VIS spectrophotometer (Shimadzu, model UV-1601 PC, Kyoto, Japan) at λmax = 290 nm.

##### 2.2.5.5. Effect of storage

For a period of 3 months and at 4 °C, the optimum formula was preserved for studying the impact of short term storage of the physical characteristics of the formula. At zero time and at the end of 3 months, the samples were withdrawn and assessed for PS, ZP, EE (%). The effect on the parameters was studied in comparison with the freshly prepared formula utilizing one-way ANOVA analysis and level (*p* < 0.05) was used to compare the significance of the results.

#### 2.2.6. Cellular study

##### 2.2.6.1. Cell culture

1% (w/v) penicillin, 1% (w/v) nonessential amino acid 10% (v/v) FBS, 1% (w/v) sodium pyruvate and streptomycin 1% (w/v) were added to MEM/EBSS and then used as growth medium for Caco-2 cells. The cells were later cultivated in 1% (w/v) streptomycin, 10% (v/v) FBS and 1% (w/v) penicillin contained in DMEM. In final, the incubation of cells was implemented with 5% CO2 supply at 37 °C and 90% relative humidity (El-Sabawi et al., 2019).

##### 2.2.6.2. Caco-2 cells cytotoxicity investigation

MTT technique was implemented for the evaluation of cytotoxic impact of the optimum formula compared with RSV dispersion. In 96-well plates, the seeding of the Caco-2 cells was performed at a density of 5 × 10 3 cells/well. (NEST Biotechnology Co. Ltd., China) then for 72 h they were cultured. The cells were subjected to (0.1, 1, 10, 100 and 1000 µg/ml) concentrations from each of the optimum formula and the drug dispersion for 12 h. Afterwards, the replacement of the medium with 200 μl of the MTT solution (0.5 mg/ml in PBS) and for 4 h the cells were incubated. The supernatant was then aspired and the formazan crystals were dissolved using dimethyl sulfoxide (200 μl/well). (ThermoFisher, Massachusetts, USA), Varioskan Flash multiplate reader was employed to assess the absorption at 570 nm and the relative cell survival was computed (Hegazy et al., 2022).

##### 2.2.6.3. Cellular uptake

In order to evaluate the uptake of the drug in cells, in 24-well plates (NEST Biotechnology Co. Ltd., Wuxi, China), the Caco-2 cells (5 × 10 3 cells per well) were seeded. Every 2 days the medium was replaced with fresh one in week 1, after that the daily change of the medium was performed until Day 14. Before the cells being incubated with 100 µg/ml of both RSV dispersion and RSV loaded optimum formula for different durations (15, 30, 60 and 120 min), for 0.5 hr, they were pretreated with freshly prepared PBS at 37 °C; for complete detailed procedure, see the Supplementary material.

#### 2.2.7. In vitro cytotoxicity and antiviral activity against SARS-CoV-2

In biological safety cabinet level 3, the titration of hCoV-19/Egypt/NRC-3/2020 isolate (SARS-CoV-2 virus) was performed using Vero-E6 cells (ATCC, CRL-1586). The Vero-E6 cells were infected in 96-well tissue culture plates utilizing serially diluted virus post confluency. In a 96-well plates, the cells were harvested and then preserved in humidified incubator under 5% CO2 at 37 °C. The cells monolayer was washed twice; consequently, the cells were then infected at 37 °C for 72 h using the serially diluted virus. The fixation of the cell monolayers was conducted using 3% paraformaldehyde followed by staining using crystal violet (0.1%). Finally, Reed and Munch equation was involved in the calculation of virus titer (Pasquereau et al., 2021).

Based on the protocol mentioned by Feoktistova et al., the cytotoxicity (CC50) was explored (Feoktistova et al., 2016), in 96-wells plate, the optimum formula and pure RSV serial dilutions were involved in treatment Vero-E6 cells monolayers. After 72 h from the exposure, the cells were subjected to fixation and staining with crystal violet and the test was completed as previously mentioned. The 50% of the harvested cells death was inspected and the relevant concentration (CC50) of each investigated samples was assessed and used for comparison with the CC50 control untreated cells.

Furthermore, also as reported Feoktistova et al., the IC50 was analyzed under biological safety level 3(Feoktistova et al., 2016). The serial dilutions of optimum formula and control RSV were admixed with equal volumes of TCID50/Ml (tissue culture infectious dose) before incubation for at 37 °C 1 h. In a 96-well tissue culture plates, 100 μL of the virus–drug mix was overlaid with Vero-E6 cell for three times then the plates were incubated at 37 °C, 5% CO2 for 72 h. 4% paraformaldehyde was utilized in fixing the cells after that by using 0.1% crystal violet, they were stained. At 570 nm, the color optical density was detected post dissolving the stain with methanol. (IC50) of each of the investigated samples were assessed and compared relative to virus control.

#### 2.2.8. Molecular modeling studies

##### 2.2.8.1. Ligand preparation, protein preparation and docking process

PubChem database was used to download reversatrol as sdf format, and subsequently was subjected to energy minimization protocol Discovery Studio (DS) 5.0 client (Accelrys). SARS‐CoV-2 Mpro structure (pdb ID: 6lu7, resolution 2.16 Å) was obtained from the protein data bank (www.rcsb.org). Docking simulations were achieved using AutoDock Vina with exhaustiveness of 8. The Mpro active site was determined with a grid box (-14.04, 17.44, 66.22) with the size 25 × 35 × 25 Å (spacing 1 Å).

## 3. Results and discussion

### 3.1. Analysis of factorial design

The consequences of various formulation factors on the characteristics of the fabricated drug delivery system can be investigated using factorial design. The investigation of each response was implemented individually and fabricated according to various order models. Herein, as shown in Table 2, the predicted R^2^ values were in rational harmony with the adjusted R^2^ for all the examined responses. A precision value (>4) was attained in almost all responses which assured the validity of the designed model to lead the design space (Zakaria et al., 2021).

**Table 2.** The outcomes of the statistical and factorial investigation of 2^3^ design and related formulae of RSV-loaded PEMLs with the predicted, observed responses and deviation percent of the Optimum formula (F5).

Responses	EE (%)	PS (nm)	ZP (mV)
R2	0.994	0.993	0.997
Adjusted R2	0.989	0.988	0.981
Predicted R2	0.976	0.974	0.825
Adequate precision	41.1	38.5	23.2
Significant factors	X1, X2, X3	X1, X2, X3	X1, X2, X3
Observed value of the optimal formula (F5)	86.1	228.9	−39.8
Predicted value of the optimal formula (F5)	84.1	227.4	−40.3
Absolute deviation %	2.35	0.65	1.25

### 3.2. Impact of fabrication factors on the characteristics of the formulated RSV-loaded bilosomes

#### 3.2.1. Influence of the fabrication factors on EE%

**Figure 1.**
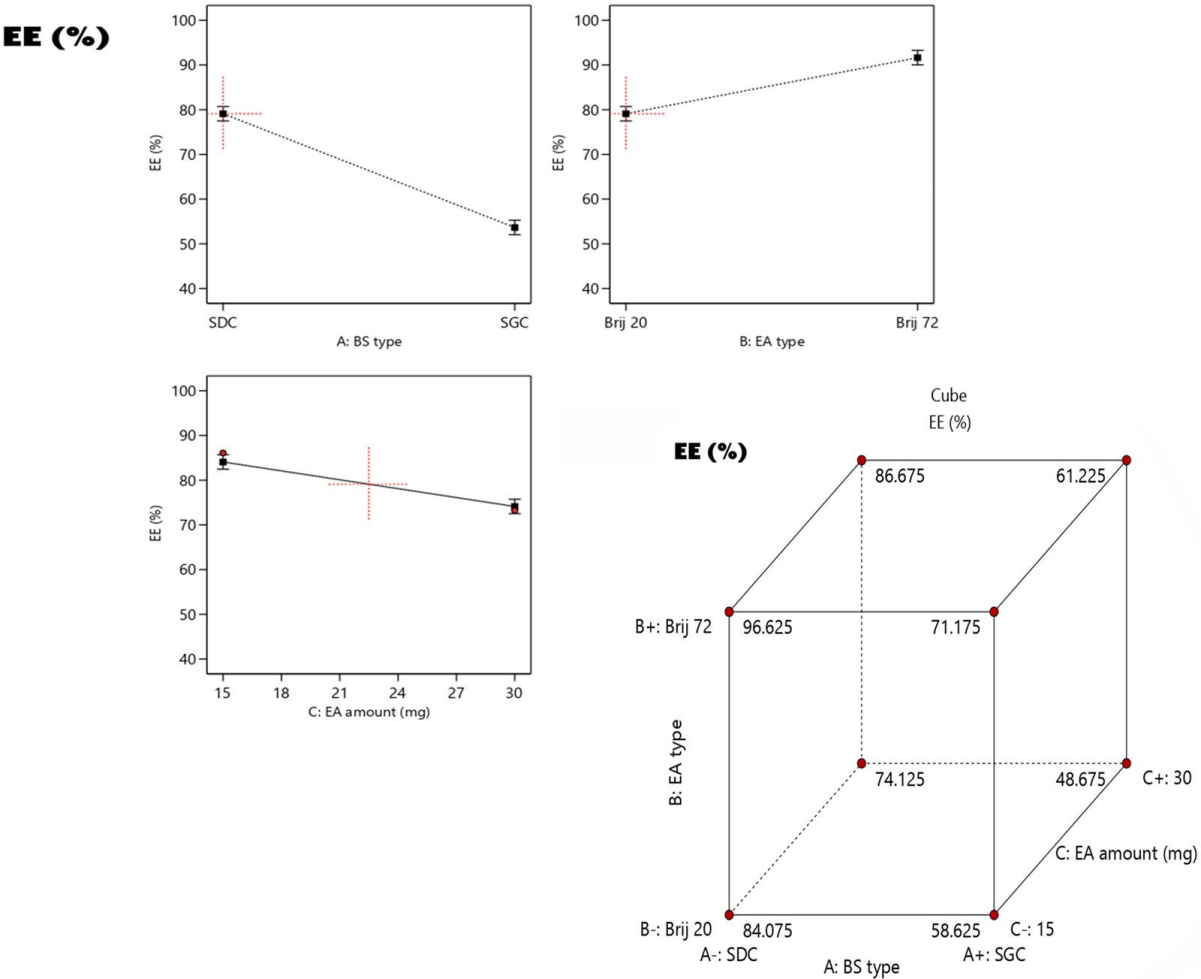
Linear and cubic plots revealing the impact of fabrication variables on EE% and Predicted versus actual correlation plot.

The endorsement of significant quantity of RSV is a crucial issue that give a hint on the potentiality of the bilosomes to be used as panel for oral delivery of the drug. As demonstrated in Table 1, the %EE varied from (47.9 ± 2.8 to 94.7 ± 4.3%). According to ANOVA analysis, it can be concluded that all the factors under investigation; A (bile salt type) factor B (EA type) and factor C (EA amount) had significant effect on the ability of the bilosomes to entrap the drug. The impacts of all factors are graphically displayed as linear and cubic plots; see Figure 1.

**Table 1.** The full factorial design adopting 2^3^ factorial analysis with the assessed responses of RSV-loaded Bilosomes.

Formula	X1 (BS type)	X2 (EA type)	X3 ( EA amount in mg)	Y1 (EE%)	Y2 (PS nm)	(PDI)	Y4 (ZP -mV)
F1	SDC	Brij 72	30	87.5 ± 1.3	225.8 ± 26.8	0.27 ± 0.04	21.8 ± 5.4
F2	SDC	Brij 72	15	94.7 ± 4.3	263.9 ± 32.8	0.34 ± 0.06	31.7 ± 1.9
F3	SGC	Brij 72	30	62.1 ± 2.1	321.4 ± 38.3	0.24 ± 0.06	40.9 ± 6.2
F4	SGC	Brij 72	15	71.4 ± 3.6	354.2 ± 51.4	0.47 ± 0.08	45.3 ± 5.2
F5	SDC	Brij 20	15	86.1 ± 2.9	228.9 ± 8.5	0.31 ± 0.08	39.8 ± 1.3
F6	SGC	Brij 20	15	58.3 ± 1.8	308.1 ± 19.7	0.62 ± 0.1	54.3 ± 3.9
F7	SDC	Brij 20	30	73.2 ± 2.4	201.2 ± 36.45	0.41 ± 0.05	34.3 ± 2.1
F 8	SGC	Brij 20	30	47.9 ± 2.8	282.6 ± 21.1	0.28 ± 0.04	50.2 ± 4.6

* N.B: 20 mg cholesterol was incorporated in each of the prepared formulae.

Taking into consideration factor A (BS type), a significant higher EE% (*p* < 0.0001) could be noticed for formulae containing SDC compared to those containing SGC and this can be explained by the higher lipophilicity of SDC (HLB = 17.6) relative to that of SGC (HLB = 23.1) which predisposed to proper intercalation of the lipophilic RSV within the hydrophobic core of the vesicles and consequently resulted in higher drug entrapment (Hegazy et al., 2022; Mosallam et al., 2021).

Concerning factor B (EA type), ANOVA results declared that the values of EE% were significantly higher (*p* = 0.0004) in formulae composed of Brij 72 relative to those composed of Brij 20. This could be justified on the basis of degree of lipophilicity, whereas Brij 72 (HLB = 4.9) is more lipophilic than Brij 20 (HLB = 15.3) led to higher entrapment of the lipophilic RSV (AbouSamra and Salama, 2017). Furthermore, the difference in the structure of each EA could significantly affect the EE%, as Brij 20 contains a double bond in its acyl chain, while Brij 70 contains no double bond. It was previously reported that a bend in the vesicular structure might be occurred as a consequence of the presence of unsaturated double-bond in the chain of carbon which results in less firmly packed and more leakier vesicles (Bnyan et al., 2018). Additionally, the lipid-phase transition temperature (Tc) of the EA have also a crucial impact on EE%, as Tc of Brij72 and Brij 20 are 44 °C and 25 °C, respectively (Abdelbary et al., 2017). As previously mentioned that EA of higher Tc will be able to construct well packed, compacted and well-ordered bilayer structure compared to EA of lower Tc that might predispose to enhanced EE% (Bnyan et al., 2018).

Also the statistical analysis revealed that doubling the EA amount from 15 mg to 30 mg resulted in a significant negative impact on the EE% (*p* = 0.001). Higher amount of EA predispose to the introduction of more pores within the vesicular structure rendering it more leaky, additionally, the fluidity of the bilayer may be also increased as a consequence of the incorporation of higher amount of EA, subsequently a decline in EE% values could be noticed (Zakaria et al., 2022a).

#### 3.2.2. Poly dispersity index (PDI) and influence of the fabrication factors on PS

**Figure 2.**
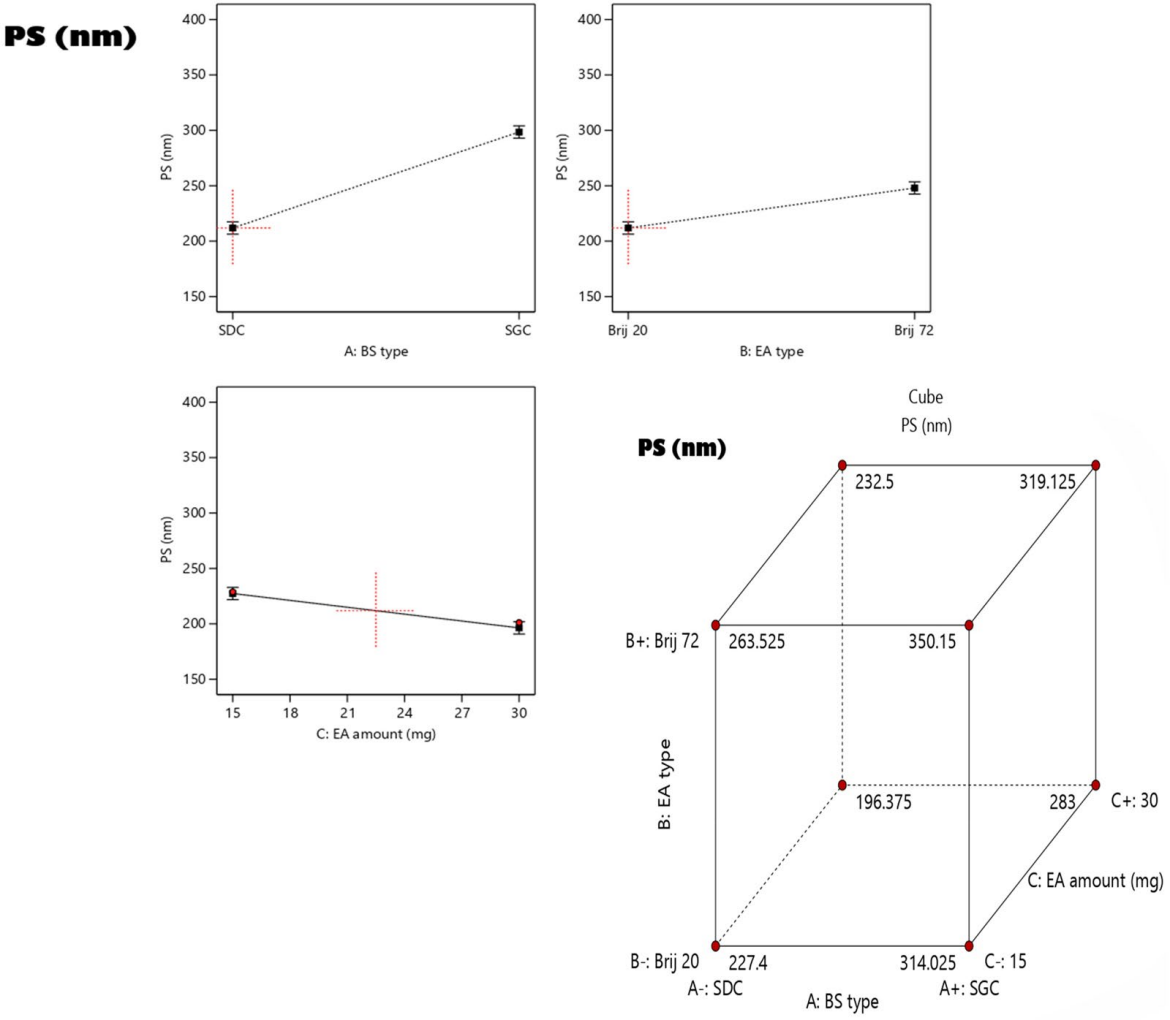
Linear and cubic plots revealing the Impact of fabrication variables on PS and predicted versus actual correlation plot.

The extent of sample homogeneity and degree of uniform dispersity can be deduced from PDI. The monodipersity can be attained with PDI values close to zero, meanwhile, as the PDI values get closer to 1 denote polydispersity. As shown in Table 1, the PDI values of the RSV-loaded bilosomes ranged from 0.24 ± 0.06 to 0.62 ± 0.1. The destiny of the drugs along with their in vivo efficacy and metabolism are critically affected by PS of the vesicles. The hindrance of the drugs detection by the complements of the blood and prolongation of their systemic circulation time can be accomplished by decreasing the PS and consequently promoting the therapeutic effects of the enclosed drugs. As illustrated in Table 1, the PS of the prepared bilosomes was in the range of 201.2 ± 36.45 to 354.20 ± 51.40 nm. According to ANOVA analysis, it can be concluded that all the factors under investigation; A (bile salt type) factor B (EA type) and factor C (EA amount) had significant effect on the PS. The impacts of all factors are graphically displayed as linear and cubic plots; see Figure 2.

Concerning factor, A (bile salt type), the formulae prepared using SDC exhibited lower PS (*p* < 0.0001) relative to those prepared using SGC and this could be justified by the level of hydrophilicity, as BS of lower hydrophilicity SDC (HLB = 17.6) than SGC (HLB = 23.1) leading to a drop in surface free energy of the system, in addition, the increase in hydrophilicity of the BS subsequently increase the water uptake within the vesicles leading to the formation of larger PS (Zaki et al., 2021). Also the increase in ZP values accompanied with SGC relative to SDC led to the formation of vesicles of larger PS owing to the increase in the gap between the vesicles resulted from the higher repulsive force between adjacent charged bilayers (El Zaafarany et al., 2010).

Furthermore, the attained results confirmed that the PS of formulae prepared using Brij 20 were significantly smaller than those of Brij 72(*p* = 0.0008). As previously mentioned, the EA of higher PEG units possesses higher steric hindrance effect and better capability to delay the precipitation of the vesicles (Brijo20 and Brij72 contain 20 and 2 PEG units), respectively (Caliceti et al., 2004). In addition, EA of higher HLB value as Brij 20 relative to that of Brij 72 will augment the surface free energy thus lower PS. Regarding the amount of EA (Factor C), it was found that increasing the amount of EA resulted in significant decline in PS (*p* = 0.0015). This can be explained by the ability of Brij to stabilize the vesicles, as on using EA with high amount was sufficient enough to wrap the vesicles and hinder their aggregation due to the steric stabilization induced by PEG units. Moreover, the surface active nature of Brij augmented in lowering the interfacial tension of the system, thus increasing its stability and resulted in formation of vesicles of lower PS. In contrary, all these effects will be diminished on using lower amounts of EA and consequently led to enlargement in PS (Abdelbary and AbouGhaly, 2015; Zakaria et al., 2022a).

#### 3.2.3. Influence of the fabrication factors on ZP

**Figure 3.**
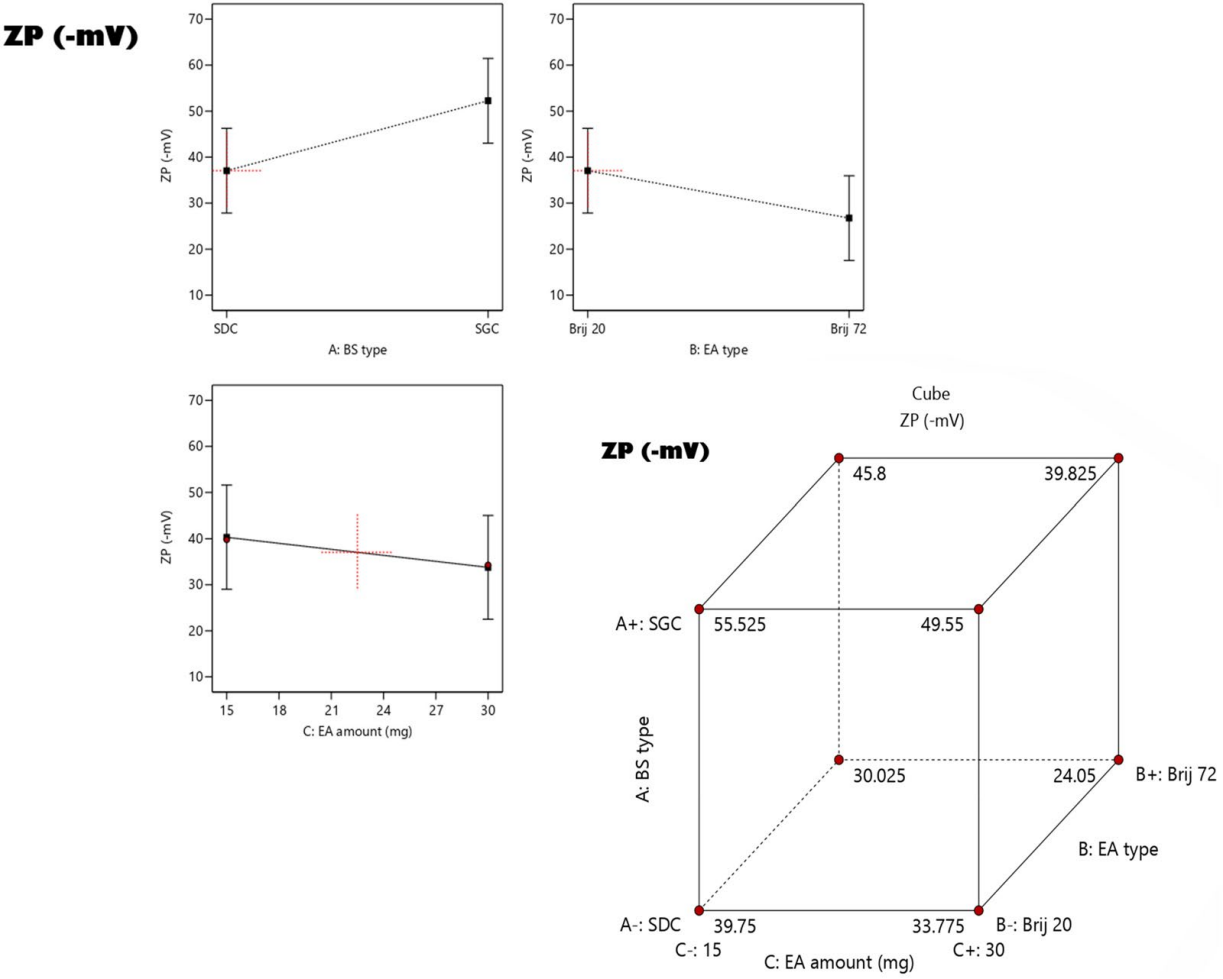
Linear and cubic plots revealing the Impact of fabrication variables on ZP and predicted versus actual correlation plot.

The electric repulsion between the vesicles based on the values of ZP denoted the stability of preparation. Originally, the ZP of system lies around ±30 mV is contemplated to be stable (Abdelbary et al., 2018). As demonstrated in Table 1 the ZP values of the prepared formulae ranged from (−21.8 ± 5.4 to −54.3 ± 3.9) affirmed that all the fabricated formulae attained enough charges to prohibit the aggregation of the particles. According to ANOVA analysis, it can be concluded that all the factors under investigation; A (bile salt type) factor B (EA type) and factor C (EA amount) had significant effect on the ZP. The impacts of all factors are graphically displayed as linear and cubic plots; see Figure 3.

Regarding factor, A (bile salt type), ANOVA results revealed that formulae prepared using SGC acquired significant (*p* = 0.0002) higher ZP values (as absolute values) than those prepared using SDC. This can be justified by the fact that the higher acidity of the glycine conjugate in SGC than the unconjugated homologues as SDC which in turn resulted in densify the negative charges on SGC containing vesicles (Bortolini et al., 2011). Moreover, higher molecular weight bile salts as SGC leads to formation of thick and dense charged interfacial layer surrounding the vesicles than the lower molecular weight as SDC (Aburahma, 2014).

On another hand, changing EA type from Brij 20 to Brij 72 resulted in significant (*p* = 0.0013) negative impact on ZP values. This may be due to the PEG moieties present in the structure of each surfactant, where Brij 20 comprises 20 PEG repeated units while Brij 72 comprises 2 units and it was previously recorded that increasing the number of anionic PEG unit leads to the localization of densified negatively charged coat of PEG surrounding the vesicles that consequently increase the repulsion force between the vesicle and rendered them segregated and stable (Cheng et al., 2018; Hegazy et al., 2022; Muthu et al., 2011). Finally increasing the amount of EA predisposed to a significant (*p* = 0.0075) decline in ZP values. This can be explored by increasing the amount of EA led them to be localized at the surface of the vesicles obscuring the negative charges of the bilosomes (Basha et al., 2013).

### 3.3. Optimum RSV-loaded bilosome formula selection

The investigation of the outcomes of the responses of the formulated 8 formulae was conducted via Design-Expert® software for the selection of the optimum formula. The desirability aims primarily to predict the optimum levels of the investigated variables and augment the picking of the optimum formula. The pre-setted criteria for the selection of the optimum formula were (accomplishing maximum EE%, maximum absolute value of ZP, and minimum PS), herein it was F5 with a desirability = 0.715. F5 exhibited an EE% of 86.1 ± 2.9%, PS of 228.9 ± 8.5 nm, and ZP of −39.8 ± 1.3 mV. Furthermore, the observed and the predicted outcomes of the responses of F5 were compared for assuring the validity and the rationality of the design. A high and adequate agreement between the observed and predicted outcomes can be depicted from the attained results Table 2.

### 3.4. In vitro investigations of the optimum RSV-loaded PEML formula

#### 3.4.1. XRD

**Figure 4.**
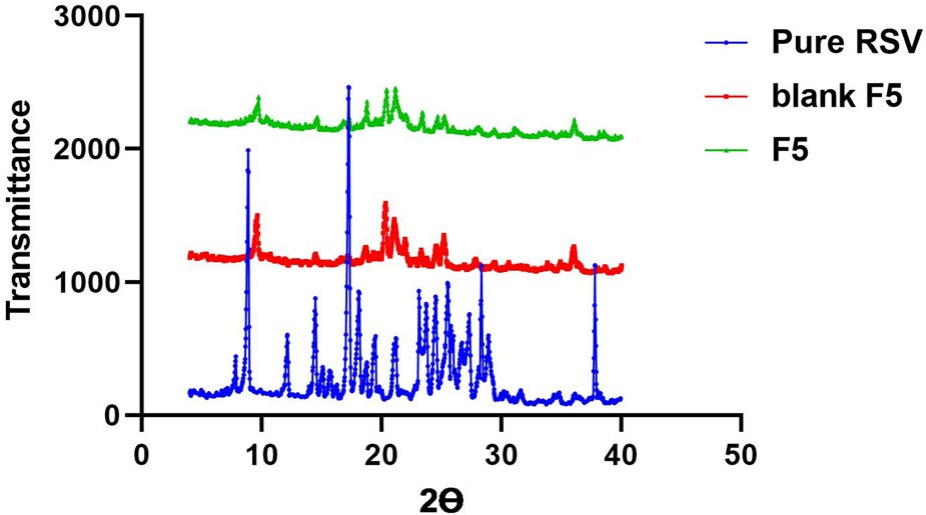
X-ray diffractogram of: Blue: pure RSV; Red and Green: blank F5 and RSV-loaded optimum formula F5.

The degree of crystallinity of a substance can be explored adopting X-ray diffraction technique. The diffraction spectra of pure RSV, blank and RSV loaded optimum formula (F5) were displayed in Figure 4. Pure RSV exhibited multiple distinct and intense peaks at 13.9, 19.5, 23.1, 25.8, 28.3 and 37.8°(Zhang et al., 2013), whereas these peaks were almost vanished in the spectra of blank and RSV loaded optimal formula (F5) denoting the conversion in the state of the drug and other components involved in formulation from crystalline state to amorphous one and affirming the efficiency of entrapment on RSV within the vesicles.

#### 3.4.2. Transmission electron microscope TEM

**Figure 5.**
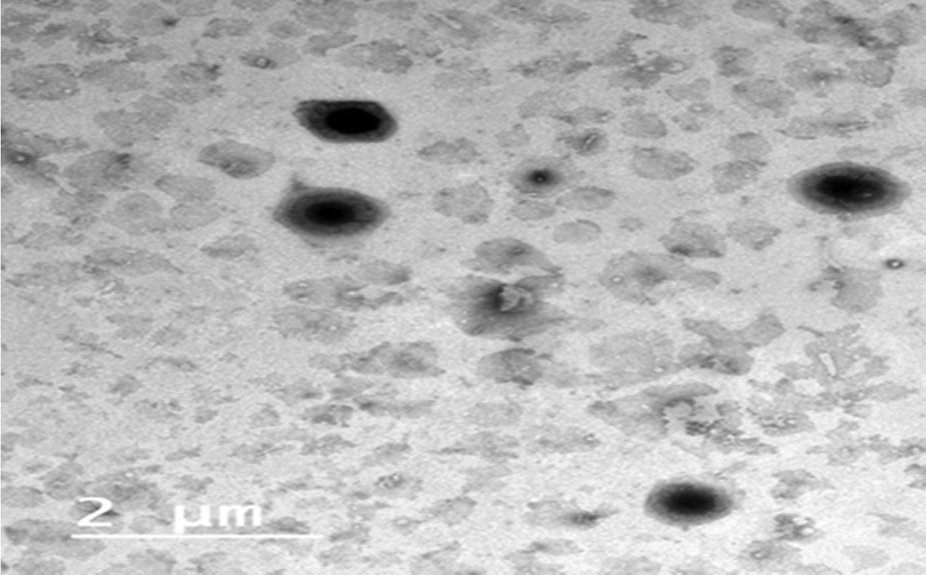
The image of TEM of the picked optimized PBs (F5)..

The TEM image of F5 declared that the vesicles exhibited round shape devoided from any abnormality Figure 5. Additionally, a fader color was noticed at the boarders of the vesicles indicating the PEG assembly at the boarders as a coat surrounding the vesicles (Zakaria et al., 2022a). The absence of any drug crystals could not be observed in the image which came in agreement with the outcome of XRD which affirm the complete inclusion RSV within the vesicles (Abdelbary et al., 2018).

#### 3.4.3. Study of RSV in vitro release

**Figure 6.**
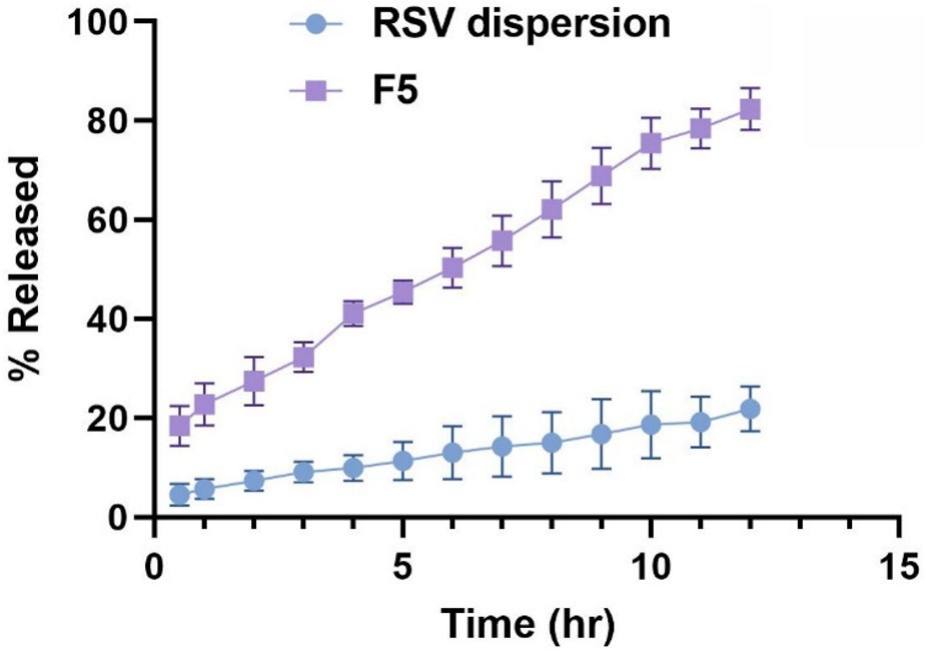
% of RSV released ± S.D. from the optimized PBs (F5) relative to that of RSV dispersion.

The amount of intact phytoceutical drug candidate released from the vesicular carrier at the target site crucially affect its therapeutic activity. Furthermore, the time, amount and location of release are highly correlated to the surface properties of the carrier system, the system composition and impact of the components of the system as surfactant, bile salt, etc. on the drug; hence, the in vitro release of the drug from the carrier system should essentially be conducted. Figure 6 revealed the release pattern of RSV from optimum formula F5 versus RSV suspension. The release of RSV form F5 exhibited successive and extended release over 12 h, where the cumulative amount released post 12 h was significantly higher and found to be 82.4 ± 3.8% compared to 21.9 ± 1.9% for RSV suspension (*p* < 0.05). This pattern could be justified by the fact that the carrier system acts as a reservoir for restricted and prolonged release of the drug followed a fast primary release phase, where the total drug released in the first 2 hours were 32.1 ± 2.2%. Meanwhile, the poor wettability and solubility of the drug may be behind the diminished release of the drug from RSV suspension. Furthermore, the presence of bile salts and the assembled PEG units of Brij as EA surrounding the vesicles predisposed to enhanced drug solubilization and higher RSV releases, as they will augment the drug solubility and increase the hydrophilicity of the system, thus the drug could be easily dispersed in the release media without any agglomeration (Alemi et al., 2018). Furthermore, it was previously reported that the PEGylation could protect the vesicles from the harsh conditions.

#### 3.4.4. The influence of storage on in vitro physical criteria of the optimal formula

From Table 3, it can be depicted that parameters under investigations: EE%, PS and ZP did not significantly affected by the short term storage of F5 for 3 months at 4 °C and at 25 °C (*p* > 0.05), owing to the steric stabilization accomplished due to the involvement PEGylated EA which act as a coat aided in prohibition of drug leakage and vesicular agglomeration, thus promoting the vesicular stability (Zakaria et al., 2021). Moreover, the high ZP value (as an absolute value) of the optimum formula which was attained from the anionic bile salt and the PEG units may also contributed in the stabilization of the vesicles and prohibiting both their aggregation and drug leakage.

**Table 3.** Storage impact on the physical criteria of the optimum RSV-loaded bilosomes (F5).

Parameter	Fresh F5	Effect of storage on F5	Effect of storage on F5
		4^o^C	25^o^C
EE%	86.1 ± 2.9	85.51 ± 1.4	83.2 ± 3.7
PS	228.9 ± 8.5	226.4 ± 10.5	221.4 ± 7.4
ZP	−39.8 ± 1.3	– 37.9 ± 0.65	−35.9 ± 4.6

### 3.5. Cellular studies

#### 3.5.1. Cytotoxicity analysis

**Figure 7.**
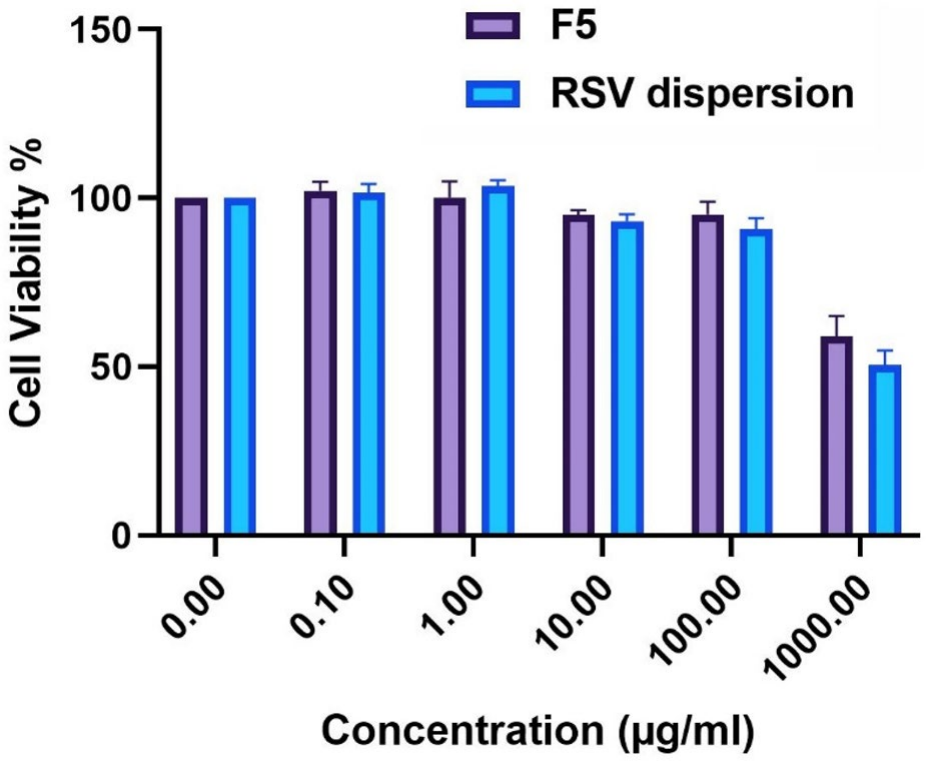
% of viability ± S.D of Caco-2 cells upon exposure to RSV-loaded F5 PBs versus the pure RSV dispersion.

The extent of cytotoxicity of tested compounds (RSV dispersion and optimum formula F5) was investigated using MTT assays. From Figure 7, it can be depicted that RSV and F5 didn’t exhibit any significant impact on Caco-2 cells viability within the tested concentration range of 0.1–100 μg/ml, thus the appropriate compatibility could be concluded. However, cell viability declined with further increase in concentration that exceeded 100 μg/ml reaching to 1000 μg/ml, where apparent cytotoxicity (*P* < 0.05) was noticed which could be justified by the presence of high amounts of SAA and bile salts concerning (F5). Finally, the 0.1–100 μg/ml was the safe concentration range used for cellular uptake assessment (Hegazy et al., 2022).

#### 3.5.2. Cellular uptake of RSV

**Figure 8.**
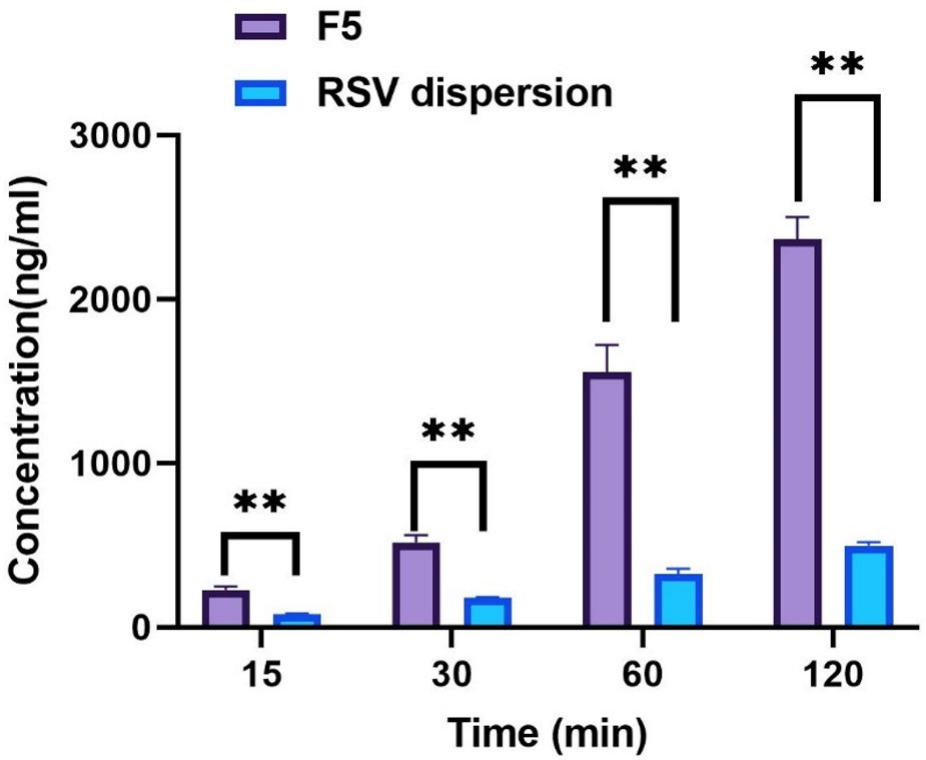
Concentration of RSV permeated within Caco-2 cells after incubation with RSV dispersion compared to optimum RSV-loaded PBs (F5) at different time intervals** Denotes significance level at *p* < 0.01.

The cellular uptake of RSV Caco-2 cells was evaluated from F5 compared to that of RSV dispersion. Figure 8 revealed that throughout the duration of experiment (120 min), the drug internalization from all samples was increased as the time elapsed. Moreover, after 2 h the uptake of RSV from F5 was significantly (*P* < 0.05) higher by around 4.7 folds than that of RSV dispersion, 2366.24 ± 135.3 ng/ml 495.65 ± 21.9 ng/ml, respectively. This significant improvement in cellular internalization after formulation was attributed to the tiny PS of the vesicles loaded with the drug, in addition to the components involved in the formulation as bile salts and surfactant exhibited a vital impact on the alteration of cellular membranes permeability (Bapat et al., 2019). Also owing to the capability of the fabricated bilosomes to conjugate either by endocytosis or by fusion to the cells may have accounted for the boosted cellular uptake of the RSV in its F5 formulation. The attachment of the PEGylated vesicles to the cellular membranes increased the concentration and consequent enhancement of the thermodynamic activity gradient of the RSV, thus promoting the permeation ability of lipophilic moieties as RSV (log *p* = 3.2) (Bapat et al., 2019; Poonia et al., 2020).

### 3.6. In vitro antiviral activity and cytotoxicity studies

#### 3.6.1. Adopting crystal violet assay in computing CC50 and IC50

**Figure 9.**
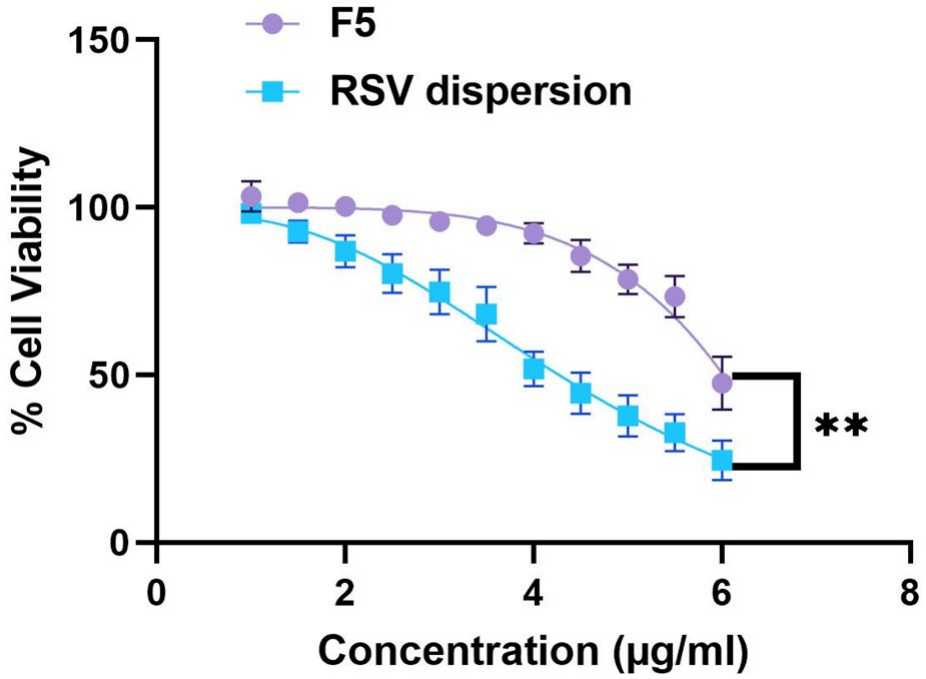
Vero-E6 cellular viability results of A: RSV dispersion (Blue); RSV-loaded PBs (F5) (Purple). *Denotes significance level at *p* < 0.05.

**Figure 10.**
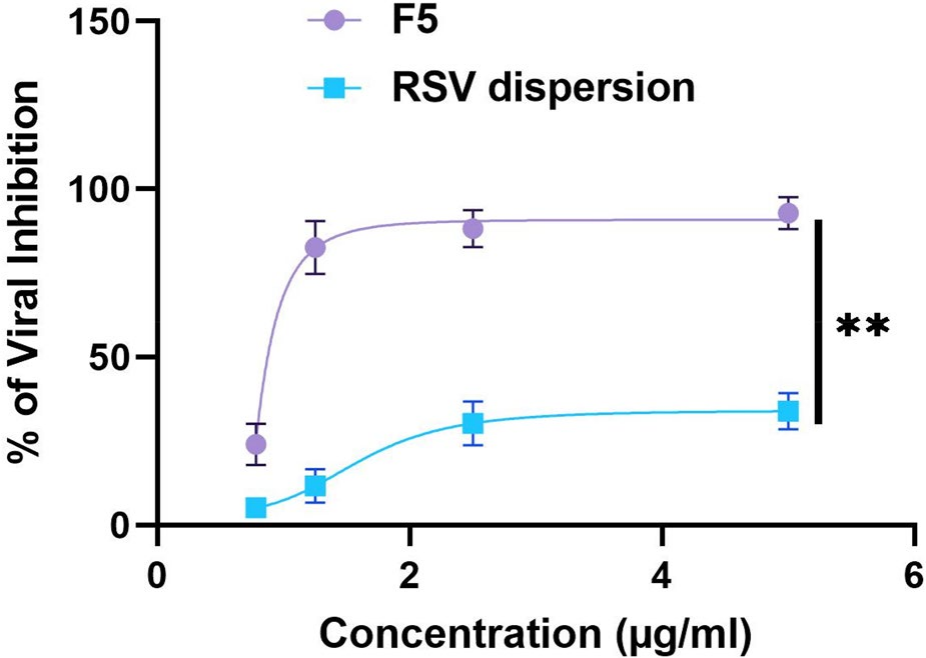
Plaque assay inhibition results of RSV dispersion (blue); RSV-loaded PBs (F5) (purple). **Denotes significance level at *p* < 0.01.

Using Vero- E6 cells, *RSV* dispersion in PBS and the optimum RSV-loaded bilosome (F5) cytotoxic effect was explored adopting MTT technique and from the results, it can be depicted that the CC50 values were 4.7 and 33.7 μg/ml, respectively (Figure 9). Based on dose–response, the RSV dispersion versus RSV-Loaded bilosome (F5) antiviral activities were assessed. The investigation of the cytotoxic effects was employed to assure that the inhibitory effect of tested samples on SARS-CoV-2 virus that caused the cell death, moreover, viral titer suppression couldn’t be considered as a consequence of host cell death, hence, the safe concentrations were involved for IC50 calculations. Figure 10 displayed the outcomes of antiviral action of RSV dispersion compared to F5. The values of the IC50 of both RSV dispersion and F5 were 1.6 and 0.24 μg/ml, respectively, with 6.6 times improvement in activity. Additionally, the ratio of CC50 to IC50 of each samples was considered for the appraisal of the selectivity indexes, where, the SI of F5 was found to be 139.5 while the SI of RSV dispersion was 2.9, declaring the significant (*p* *<* 0.05) augmentation of bilosomal formulation in the activity of F5. The aforementioned outcomes revealed that *RSV* formulation using PEGylated bilosomal system attained the capability of boosting the antiviral activity and promoting the clinical of *RSV*, stunningly, at a minute concentration reached to 0.48 μg/ml, the complete suppression of the virus was achieved.

### 3.7. Molecular modeling study

**Figure 11.**
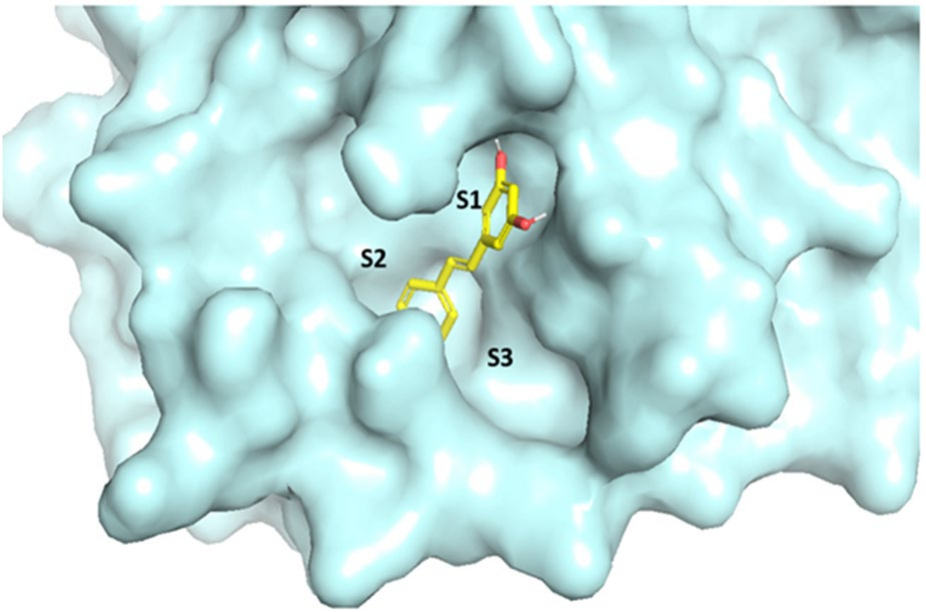
Surface view of the binding mode of resveratrol into active site of SARS-CoV-2 Mpro. S1, S2 and S3 are the main binding subunits.

It was obvious that resveratrol could pertain its antiviral activity by significantly diminishing the proteolytic activity of SARS-CoV-2 main protease (SARS-CoV-2 Mpro) (Jo et al., 2019). For that reason, this molecular docking study was conducted on SARS-CoV-2 Mpro to explain the outstanding antiviral activity of resveratrol. AutoDock Vina was used to accomplish the docking simulation (Trott and Olson, n.d.). Determination of the binding affinities to the binding pockets of SARS-CoV-2 Mpro was configured through formation of crucial H-bonds and/or hydrophobic contacts with catalytic Cys145-His41 dyad within the active site of S1 binding site (Dai et al., 2020; Jin, 2020; Rathnayake et al., 2020), alongside the binding energy scores. Validation of Auto Dock Vina docking protocol afforded by redocking Mpro co-crystallized ligand, where excellent coexistence between crystallographic N3 ligand and the best fitted redocked pose was noticed with RMSD = 0.56 A. Domains I and II construct the Mpro catalytic site that it is flanked by a dyad Cys145 and His41. Moreover, it was noticed that the key residues Phe140, Leu141, Asn142, Gly143, Ser144, Met165, Glu166, Gln189, and Thr190 compose the Mpro binding pocket (Dai et al., 2020; Jin, 2020; Rathnayake et al., 2020). The docking simulations of the Mpro active site indicated that resveratrol located on the S1, S2 and S3 of the binding site with binding free energy of −7 kcal/mol, where resorcinol (m-dihydroxyphenol) fragment of resveratrol occupied S1 subunit and the phenol fragment occupied S2 subunit (Figure 11).

**Figure 12.**
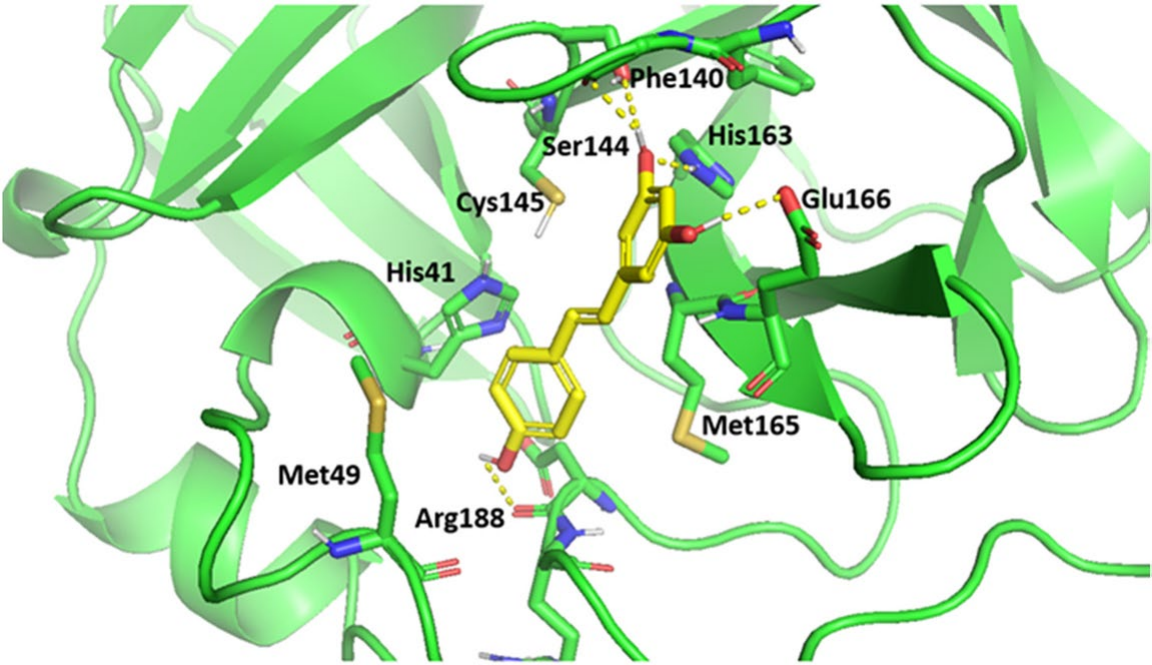
3 D structural models of resveratrol into the active site of SARS-CoV-2 Mpro. The H-bond interactions are represented by dotted lines.

In depth, resveratrol is perfectly stuck to the S1 binding site (Figure 12), where a network of 3 H-bonds (Phe140, Ser144 and His163) that stabilize resveratrol into the active site of enzyme were mapped. Moreover, a crucial H-bond was formed between Glu166 of S3 subunits and the resorcinol hydroxyl group. Alongside, H-bond between Arg188 and phenol OH moiety stabilizes resveratrol-receptor complex. Additionally, the critical pi-cation interaction between the resorcinol fragment of resveratrol and the catalytic Cys145 was configured. The ethenyl phenol fragment is also flanked by the side chains of His41, Met49, Met165, Asp187, and Arg188, affording massive hydrophobic contacts within the Mpro pocket. The π-stacking hydrophobic interaction between His41 and resveratrol (Sies and Parnham, 2020) besides, the hydrophobic interactions with Met49, His41, and Met165 residues (Gurung et al., 2020) and H-bonds with Ser144 play an essential role in the protease-inhibitor complex stabilization in SARS-CoV-2 Mpro (Stoddard et al., 2020). This docking simulation study steadily demonstrated that the outstanding inhibitory activity of resveratrol could be attributed to diminishing the proteolytic activity of SARS-CoV-2 Mpro.

## 4. Conclusion

In the conducted study, PBs were fabricated adopting 2^3^ full factorial experiment for oral delivery of RSV. F5 was sorted out as optimum formula with desirability value 0.707. Moreover, F5 exhibited spherical shape with high drug EE%, ZP and small PS. Additionally, Caco-2 cells permeability study revealed the superiority of F5 over RSV dispersion by around 4.7 folds’ increase in cellular uptake. Stunningly, F5 exhibited enhanced antiviral activity against SARS-CoV-2 by 6.6 folds relative to RSV dispersion. The conducted modeling study revealed that resveratrol is perfectly oriented toward the catalytic Cys145-His41 dyad within the active site of main protease. Consequently, resveratrol pertains high affinity to prohibit the SARS-CoV-2 Mpro enzyme. Finally, the aforementioned outcomes affirmed that F5 was capable of overcoming its severe first-pass metabolism and other oral problems associated with its oral administration. Thus, F5 could be proposed as a potential carrier for oral delivery RSV with accentuated anti-SARS-CoV-2 activity.

## Supplementary Material

Supplemental Material
