# Supplementary material for: ‘Poly phenolic phytoceutical loaded nano-bilosomes for enhanced caco-2 cell permeability and SARS-CoV 2 antiviral activity’: in-vitro and insilico studies
Source: Drug Deliv. 2023 Jan 1;30(1):2162157. doi: 10.1080/10717544.2022.2162157 (PMC9809390; doi:10.1080/10717544.2022.2162157)
Supplement: Supplemental Material [file IDRD_A_2162157_SM7871.docx]

**Supplementary Material**

Caco-2 cells (5 ×10 3 cells per well) were seeded in 24-well plates (NEST Biotechnology Co. Ltd., Wuxi, China) to evaluate cellular uptake. In Week 1, the medium was changed every 2 d The medium was refreshed daily until Day 14. The Caco-2 cells

were then pretreated with fresh PBS at 37 °C for 0.5 h. The cells were cultured with 100 μM of the RSV suspension, F5 for different durations (0.25, 0.50, 1.00, and 2.00 h). The Caco-2 cells were subsequently washed with PBS and collected with 300 μl of water. The samples were disrupted in the SCIENTZ-IID Ultrasonic Homogenizer (Ningbo Scientz Biotechnology Co., Ltd., Ningbo, Zhejiang). Total protein was quantified using a BCA assay kit. To analyze the amount of, the samples were hydrolyzed by β-glucuronidase . About 200 μl of the cell sample was first deproteinized by vortexing with 600 μl of ethanol and then centrifuged 11 000 *g* for 5 min. The supernatant was dried and resuspended with PBS. The pH of the sample was subsequently adjusted to 4.5–5.5 and incubated with 25 μl β-glucuronidase (sodium acetate buffer, 30 mg/ml) and 12.5 μl of ascorbic acid (10% *w/v* ) in the dark at 37 °C for 1 h. The samples were then incubated with ethyl acetate, followed by vortexing for 5 min. After centrifugation at 11 000 *g* for 5 min, the supernatant was collected, evaporated, redissolved in methanol, and determined by HPLC
